# Reviewers

**DOI:** 10.1111/cas.15192

**Published:** 2021-12-05

The publication of invaluable papers in *Cancer Science* depends on the prompt, careful review of submitted manuscripts. We would like to thank the following experts for reviewing manuscripts submitted between October 1, 2020 and September 30, 2021.

Wajid Arshad Abbasi

Hiroyuki Abe

Masahiro Abe

Tatsuya Abe

Jun Adachi

Souichi Adachi

Hiroshi Aikata

Shinichi Aishima

Kiwamu Akagi

Shusuke Akamatsu

Takumi Akashi

Yoshiki Akatsuka

Hiroshi Akazawa

Hideaki Akita

Yoshimitu Akiyama

Kiyohiro Ando

Mizuo Ando

Kenjiro Aogi

Kazunori Aoki

Shuichi Aoki

Yoshiki Arakawa

Mitsugu Araki

Nobuhito Araki

Takaaki Arigami

Hajime Asahina

Ayumu Asai

Yoshinari Asaoka

Eishi Ashihara

Norio Asou

Shinji Atagi

Haruhito Azuma

Eishi Baba

Hideo Baba

Masaya Baba

Tomohisa Baba

Tsukasa Baba

Yoshifumi Baba

Arup Bag

Hideaki Bando

Kouji Banno

Antonio Barbieri

Giovanni Blandino

Vaclav Brazda

Horacio Cabral

Marco Candeias

Yihai Cao

Kenji Chamoto

Eric Chan

Ying Chen

Zhe‐Sheng Chen

Natsuko Chiba

Tetsuhiro Chiba

Kazuaki Chikamatsu

Mariko Chikuma

Shunsuke Chikuma

Tatsuyuki Chiyoda

Makoto Chuma

Yoshiro Chuman

Shingo Dan

Arvind Dasari

Kaori Denda‐Nagai

Ning Deng

Xiaozhao Deng

Takahiro Domoto

Hiromichi Ebi

Hidetaka Eguchi

Hidetoshi Eguchi

Susumu Eguchi

Shogo Ehata

Makoto Endo

Hideki Enokida

Keisuke Enomoto

Yutaka Enomoto

Valerio Farfariello

Nuno Figueiredo

Isabel Fonseca

Thierry Frebourg

Li Fu

Shigeo Fuji

Hideki Fujii

Hirofumi Fujii

Satoshi Fujii

Shin‐Ichiro Fujii

Takuma Fujii

Yoichi Fujii

Akihiro Fujimoto

Kiyohide Fujimoto

Naohiro Fujimoto

Shin Fujisawa

Mitsuhiro Fujishiro

Teruaki Fujishita

Kazutoshi Fujita

Ken‐Ichi Fujita

Masatoshi Fujita

Yasuyuki Fujita

Yu Fujita

Hiroshi Fujiwara

Naoto Fujiwara

Yutaka Fujiwara

Tatsuo Fukagawa

Akihisa Fukuda

Koji Fukuda

Tomohiko Fukuda

Shota Fukuoka

Noriyoshi Fukushima

Satoshi Fukushima

Takuya Fukushima

Takashi Fukuyama

Yosuke Funato

Tatsuhiko Furukawa

Toru Furukawa

Yusuke Furukawa

Mikio Furuse

Fumihiko Furuya

Yasutaka Fushimi

Mitsuru Futakuchi

Manabu Futamura

Xingchun Gao

Min Gi

Shannon Glaser

Gary S. Goldberg

Yan Gong

Zhaohui Gong

Akiteru Goto

Hidemasa Goto

Yusuke Goto

Susumu Goyama

Eric Grant

Jianguo Gu

Jiwei Guo

Peng Guo

Akinobu Hamada

Shin Hamada

Ryuji Hamamoto

Junzo Hamanishi

Chisato Hamashima

Arum Han

Megumi Hara

Yasuaki Harabuchi

Hironori Harada

Hiroshi Harada

Mamoru Harada

Tetsunari Hase

Takahiro Hasebe

Naoya Hashimoto

Shinichi Hashimoto

Rintaro Hashizume

Hisashi Hasumi

Yutaka Hata

Shigetsugu Hatakeyama

Shingo Hatakeyama

Yuichiro Hatano

Yoshihiro Hatta

Naoko Hattori

Fumihiko Hayakawa

Yoku Hayakawa

Hidetoshi Hayashi

Yoshinori Hayashi

Shoichi Hazama

Jie He

Yasuhiro Hida

Fumi Higuchi

Hayato Hikita

Shinjiro Hino

Yoshitaka Hippo

Nobuyoshi Hiraoka

Akira Hirasawa

Eishu Hirata

Kenji Hirata

Kouyuki Hirayasu

Yamamoto Hirofumi

Yoshihiko Hirohashi

Shuichi Hironaka

Yuichi Hirose

Kiichi Hirota

Toru Hirozane

Asahi Hishida

Teruyoshi Hishiki

Takaki Hiwasa

Chao‐Chi Ho

Robert Hoffman

Hiroaki Honda

Masao Honda

Takayuki Honda

Koichi Honke

Masaru Honma

Hidehito Horinouchi

Keisuke Horiuchi

Naoki Hosen

Takayuki Hoshii

Ayuko Hoshino

Masahito Hosokawa

Naoko Hosono

Satoyo Hosono

Yasuyuki Hosono

Katsuyuki Hotta

Chao‐Kai Hsu

Gang Huang

R. Stephanie Huang

Ruby Huang

Daisuke Ichikawa

Masashi Idogawa

Tatsushi Igaki

Tsukasa Igawa

Yoshito Ihara

Daisuke Iizuka

Hideaki Ijichi

Takeshi Ijuin

Kazuhiro Ikeda

Toru Ikegami

Tsuneo Ikenoue

Takayuki Ikezoe

Yusuke Imagawa

Takeharu Imai

Toshio Imai

Yoichi Imai

Chiyo Imamura

Tatsuhiko Imaoka

Teruo Inamoto

Yasushi Inou

Akira Inoue

Daichi Inoue

Jun Inoue

Keiji Inoue

Yasumichi Inoue

Takashi Inozume

Masafumi Inui

Noriyoshi Iriyama

Hideyuki Ishida

Yusuke Ishida

Takaya Ishihara

Naoto Ishii

Yuichi Ishikawa

Naozumi Ishimaru

Takatsugu Ishimoto

Akira Ishisaki

Toru Ishitani

Kenji Ishitsuka

Kenichi Ishizawa

Takeshi Isobe

Eisuke Itakura

Hiroaki Itamochi

Naoki Itano

Akihiko Ito

Akihiro Ito

Kazuto Ito

Kosei Ito

Masanori Ito

Takahiro Ito

Tetsuhide Ito

Yoshikazu Ito

Yuri Ito

Fumio Itoh

Shinji Itoh

Susumu Itoh

Manabu Iwadate

Atsushi Iwama

Eiji Iwama

Takayuki Iwamoto

Motoki Iwasaki

Takeshi Iwasaki

Masaaki Iwatsuki

Kouji Izumi

Shunji Izuta

Koji Izutsu

Wen‐Shan Jian

Xingming Jiang

Ya‐Qiong Jin

Keiichi Jingu

Chamila Chathuranga Kadigamuwa

Norimitsu Kadowaki

Takayuki Kadoya

Hidenori Kage

Shinichi Kageyama

Yuki Kagoya

Kyoichi Kaira

Tsuneyasu Kaisho

Taisuke Kajino

Kentaro Kajiwara

Hiroaki Kajiyama

Anna Kakehashi

Yoshihiro Kakeji

Kazuhiro Kakimi

Nobuyuki Kakiuchi

Takao Kamai

Yoichiro Kamatani

Yasuhiko Kamikubo

Naoko Kamiya

Miki Kamiyama

Takayuki Kanaseki

Hiroaki Kanda

Teru Kanda

Yoshinobu Kanda

Hirokazu Kanegane

Shuzo Kaneko

Yasuhiko Kaneko

Chang Moo Kang

Mitsunobu Kano

Yoshihito Kano

Yoshinobu Kariya

Christiana Kartsonaki

Kennosuke Karube

Kazuo Kasahara

Hitoshi Kasai

Kota Katanoda

Hiroaki Kataoka

Keisuke Kataoka

Kazuhiro Katayama

Ken Kato

Kikuya Kato

Koji Kato

Mamoru Kato

Motohiro Kato

Takuya Kato

Yukinari Kato

Hiroto Katoh

Yuki Katoh

Keisuke Katsushima

Shinji Kawabata

Tsuyoshi Kawabata

Asuka Kawachi

Manabu Kawada

Norifumi Kawada

Hiroyuki Kawahara

Hitomi Kawai

Masaaki Kawai

Taketo Kawai

Hisato Kawakami

Masanori Kawakami

Kei Kawana

Yoshihiro Kawasaki

Atsunari Kawashima

Akihito Kawazoe

Masahito Kawazu

Tanaka Kazuhiro

Hino Keisuke

Hirotsugu Kenmotsu

Ikramy Khalil

Hiroyasu Kidoya

Eiji Kikuchi

Eiki Kikuchi

Yoshikane Kikushige

Won Seog Kim

Go Kimura

Hideharu Kimura

Hiroshi Kimura

Takahiro Kimura

Tomokazu Kimura

Ichiro Kinoshita

Keita Kirito

Hiroshi Kitagawa

Masatoshi Kitagawa

Hiroshi Kitajima

Shojiro Kitajima

Shunsuke Kitajima

Sho Kitamoto

Hidemitsu Kitamura

Hiroshi Kitamura

Masayuki Kitano

Shigehisa Kitano

Joji Kitayama

Katsuyuki Kiura

Etsuko Kiyokawa

Shinichi Kiyonari

Yuko Kiyosue

Naomi Kiyota

Kazuma Kiyotani

Yoshiyuki Kizawa

Hiroshi Kobayashi

Hiroya Kobayashi

Hisataka Kobayashi

Makoto Kobayashi

Miho Kobayashi

Susumu Kobayashi

Takashi Kobayashi

Yukio Kobayashi

Yuta Kochi

Vitaly Kochin

Takahiro Kodama

Yoshikatsu Koga

Takahiro Kogawa

Yasuhiro Koh

Kenichi Kohashi

Shinji Kohsaka

Kensuke Koike

Kensuke Kojima

Takahiro Kojima

Yu‐Ichiro Koma

Hirokazu Komatsu

Norio Komatsu

Shuhei Komatsu

Yoshito Komatsu

Keigo Komine

Yoshihiro Komohara

Noritaka Komune

Daisuke Komura

Shunsuke Kon

Eisaku Kondo

Masashi Kondo

Satoru Kondo

Takashi Kondo

Tsunenori Kondo

Akimitsu Konishi

Hideyuki Konishi

Hirotaka Konishi

Masamitsu Konno

Chihaya Koriyama

Nobuyoshi Kosaka

Takeo Kosaka

Naohiko Koshikawa

Ai Kotani

Masashi Koto

Junji Koya

Shohei Koyama

Toshiyuki Kozuki

Sebastian Krug

Keiji Kuba

Terufumi Kubo

Toshio Kubo

Yasushi Kubota

Aya Kuchiba

Yasusei Kudo

Iwao Kukimoto

Rajiv Kumar

Shoen Kume

Kazuhiro Kunimasa

Kei Kunimasa

Hiroyoshi Kunimoto

Hiroki Kuniyasu

Takayasu Kurata

Naoya Kuriyama

Sei Kuriyama

Junya Kuroda

Masahiko Kuroda

Tetsuji Kurokawa

Hiroya Kuroyanagi

Kazuhiko Kurozumi

Shigeru Kusumoto

Satoru Kyo

En‐Min Li

Minmin Liang

Weijia Liao

Wan‐Teck Lim

Changwei Lin

Chia‐Chi Lin

Rong Liu

Shanrong Liu

Xiaoxia Liu

Zeyi Liu

Kwok Wai Lo

Siew‐Kee Low

Jun Lu

You Lu

Shiwen Luo

Shuhong Luo

Artem Lysenko

Mitsuhiro Machitani

Daichi Maeda

Osamu Maeda

Tomoya Maeda

Tomohiko Maehama

Masato Maekawa

Shinya Maekawa

Makoto Maemondo

Nako Maishi

Hideki Makinoshima

Hideki Makinoshoma

Yoshinori Marunaka

Reo Maruyama

Tetsuo Mashima

Kiyoshi Masuda

Muneyuki Masuda

Norikazu Masuda

Shinobu Masuda

Takaaki Masuda

Yohei Masugi

Kenta Masui

Takashi Masuko

Hiroshi Masutani

Mitsuko Masutani

Daisuke Matsubara

Mari Matsuda

Tomohiro Matsuda

Yoko Matsuda

Nobuhisa Matsuhashi

Hirotaka Matsui

Makoto Matsui

Hiroaki Matsumoto

Koji Matsumoto

Kunio Matsumoto

Masaki Matsumoto

Shingo Matsumoto

Shinji Matsumoto

Tomonori Matsumoto

Keisuke Matsusaka

Hirokazu Matsushita

Keiko Matsuura

Hideyasu Matsuyama

Juntaro Matsuzaki

Reiko Matsuzawa

Manasi Mayekar

Mary F McMullin

Yutaka Midorikawa

Shuji Mikami

Hiroaki Miki

Yasuhiro Miki

Li Min

Yosuke Minami

Yoji Minamishima

Toshinari Minamoto

Kiyoshi Misawa

Yuichi Mitobe

Norisato Mitsutake

Koh Miura

Masahiko Miura

Satoru Miura

Yutaka Miura

Yohei Miyagi

Yoshihiro Miyahara

Hideaki Miyake

Tatsuo Miyamoto

Toshihiro Miyamoto

Minoru Miyashita

Kanjiro Miyata

Shin‐Ichi Miyatake

Shiro Miyayama

Kana Miyazaki

Keisuke Miyazawa

Hiroaki Miyoshi

Norikatsu Miyoshi

Tomoichiro Miyoshi

Yasuo Miyoshi

Atsushi Mizokami

Yusuke Mizukami

Eishiro Mizukoshi

Kei Mizuno

Nobumasa Mizuno

Ryuichi Mizuno

Tsunekazu Mizushima

Kiyohito Mizutani

Fumiyasu Momose

Isao Momose

Shuji Momose

Toshimitsu Momose

Yukihide Momozawa

Seiichi Mori

Michihisa Moriguchi

Masato Morikawa

Yuji Morine

Kazuhiro Morishita

Akinori Morita

Takuya Moriya

Chigusa Morizane

Toshiro Moroishi

Sei‐Ichiro Motegi

Shinichiro Motohashi

Noriko Motoi

Ken‐Ichi Mukaisho

Toru Mukohara

Wataru Munakata

Junko Murai

Takashi Murakami

Yoshiki Murakumo

Masashi Muramatsu

Daisuke Muraoka

Takahiko Murayama

Taisei Mushiroda

Manabu Muto

Michihiro Mutoh

Kazuki Nabeshima

Genta Nagae

Koji Nagafuji

Masayuki Nagahashi

Hirokazu Nagai

Hiroaki Nagano

Osamu Nagano

Kazunori Nagasaka

Hideko Nagasawa

Satoshi Nagayama

Hisamichi Naito

Yoichi Naito

Yuji Naito

Kazuhito Naka

Shinichiro Nakada

Hironori Nakagami

Hayato Nakagawa

Makoto Nakagawa

Masao Nakagawa

Takuya Nakagawa

Tohru Nakagawa

Tomomi Nakahara

Noboru Nakaigawa

Hiroo Nakajima

Takahiro Nakajima

Yasunari Nakamoto

Eijiro Nakamura

Katsumasa Nakamura

Naoya Nakamura

Takashi Nakamura

Tsutomu Nakamura

Yoshiaki Nakamura

Eiji Nakano

Yoshiko Nakano

Takashi Nakaoku

Masahiro Nakashima

Fumihiko Nakatani

Koh Nakayama

Robert Nakayama

Yozo Nakazawa

Takeshi Namekawa

Yasuhito Nannya

Katsuhiko Naoki

Hiroto Narimatsu

Shintaro Narita

Yoshitaka Narita

Atsushi Natsume

Seiji Niho

Atsushi Niida

Hiroyuki Niida

Naoki Niikura

Toshiro Niki

Hironori Ninomiya

Jun Nishida

Mikako Nishida

Shozo Nishida

Hiroshi Nishihara

Ryuichi Nishii

Yuji Nishikawa

Momoko Nishikori

Kazuo Nishimura

Noriyuki Nishimura

Tatsunori Nishimura

Hiroshi Nishina

Soji Nishina

Hitomi Nishinakamura

Kazumi Nishino

Yoshikazu Nishino

Hiroshi Nishio

Nobuhiro Nishio

Yasuhiko Nishioka

Michiru Nishita

Noyuki Nishiya

Hiroyuki Nishiyama

Naotaka Nishiyama

Satoshi Nishizuka

Hiroyuki Nobusue

Nobuo Noda

Satoshi Noda

Kohji Noguchi

Rei Noguchi

Takuro Noguchi

Hiroshi Nokihara

Fumio Nomura

Hiroyuki Nomura

Sachiyo Nomura

Kisato Nosaka

Kaname Nosaki

Wataru Obara

Yuki Obata

Mikako Ogawa

Takenori Ogawa

Hirokazu Ogino

Hisakazu Ogita

Hideaki Ogiwara

Michinori Ogura

Takashi Ohama

Kadoaki Ohashi

Shigeo Ohba

Tomokazu Ohishi

Yoshihiro Ohishi

Fumiharu Ohka

Takayuki Ohkuri

Tohru Ohmori

Masaki Ohmuraya

Nobumichi Ohoka

Kiyoshi Ohtani

Masayuki Ohtsuka

Takao Ohtsuka

Sumio Ohtsuki

Satoshi Oizumi

Hidenori Ojima

Atsushi Okabe

Seiji Okada

Tomoko Okada

Isamu Okamoto

Shinji Okano

Akihiko Okayama

Keito Okazaki

Yasumasa Okazaki

Eiji Oki

Yukari Okita

Chio Okuyama

Chitose Oneyama

Takashi Onoe

Mamiko Onuki

Aikseng Ooi

Nobuhiko Oridate

Akira Orimo

Makoto Osanai

Motomi Osato

Takahiro Osawa

Tsuyoshi Osawa

Hiroko Oshima

Shozo Osumi

Kensuke Otsuka

Motoyuki Otsuka

Rongying Ou

Kota Ouchi

Naohide Oue

Masaaki Oyama

Shuji Ozaki

Hiroaki Ozasa

Yuuki Ozato

Jeffrey Parvin

Rosanna Piccirillo

Karol Polom

Wan Qianyi

Samuel Rabkin

Aileen G. Rowan

Bise Ryoma

Hisataka Sabe

Mahito Sadaie

Guiseppe Saglio

Yuriko Saiki

Eiko Saito

Motonobu Saito

Ryuta Saito

Takuro Saito

Yasuhiro Saito

Yasuyuki Saito

Yoshimasa Saito

Masao Saitoh

Noriko Saitoh

Akira Sakai

Hideki Sakai

Kazuko Sakai

Emiko Sakaida

Jun Sakakibara‐Konishi

Kei Sakamoto

Naoya Sakamoto

Shinichi Sakamoto

Takeharu Sakamoto

Shingo Sakashita

Mamiko Sakata‐Yanagimoto

Tomohisa Sakaue

Yukimi Sakoda

Keiichiro Sakuma

Yuji Sakuma

Hiroaki Sakurai

Hiroyuki Sakurai

Oltea Sampetrean

Masashi Sanada

Takaomi Sanda

Daisuke Sano

Tetsuro Sasada

Toshiyuki Sasagawa

Keisuke Sasai

Koji Sasaki

Nobunari Sasaki

So‐Ichiro Sasaki

Takaaki Sasaki

Tomonari Sasaki

Yasushi Sasaki

Yuka Sasaki

Megumi Sasatani

Takashi Sasayama

Goro Sashida

Mitsuo Sato

Shinya Sato

Tsutomu Sato

Yasuharu Sato

Yusuke Sato

Taroh Satoh

Akira Satou

Kenjiro Sawada

Norie Sawada

Jeffrey Schlom

Masahiro Seike

Ken‐Ichiro Seino

Naohiko Seki

Ikuo Sekine

Keisuke Sekine

Kentaro Semba

Hiroshi Seno

Tsukasa Seya

John Seymour

Aya Shiba

Kiyotaka Shiba

Yukiko Shibahara

Akiko Shibata

Atsushi Shibata

Hirohiko Shibayama

Kazuko Shibuya

Kotaro Shide

Tadahiko Shien

Kiyoto Shiga

Takashi Shiina

Kazuyuki Shimada

Midori Shimada

Shu Shimada

Teppei Shimamura

Hiroaki Shime

Kosuke Shimizu

Masahito Shimizu

Ritsuko Shimizu

Takatsune Shimizu

Toshio Shimizu

Kazuya Shimoda

Hideki Shimodaira

Akihiko Shimomura

Yoshiharu Shinden

Kato Shinichiro

Keiko Shinjo

Daisuke Shiokawa

Goshi Shiota

Masaki Shiota

Bunsyo Shiotani

Yoshiyuki Shioyama

Atsushi Shiozaki

Ken Shirabe

Kenshiro Shiraishi

Kouya Shiraishi

Yuichi Shiraishi

Toshiro Shirakawa

Sunao Shoji

Abdullah Shopit

Takehito Shukuya

Alena Skalova

Yasushi Soda

Kenzo Soejima

Junichi Soh

Kenzo Sonoda

Yukihiko Sonoda

Yoshihiro Sowa

Fu‐Hsiung Su

Kenichi Suda

Yoshiyuki Suehara

Eisaburo Sueoka

Tamotsu Sugai

Kokichi Sugano

Keishi Sugimachi

Kenji Sugio

Hiroshi Sugiyama

Hidetoshi Sumimoto

Kuniko Sunami

Masumi Suzui

Ayako Suzuki

Fumitaka Suzuki

Hiromichi Suzuki

Hiroshi Suzuki

Hiroyuki Suzuki

Kazuhiro Suzuki

Keiji Suzuki

Miho Suzuki

Motoshi Suzuki

Norio Suzuki

Shigeaki Suzuki

Susumu Suzuki

Takafumi Suzuki

Takashi Suzuki

Takeshi Suzuki

Yoshiyuki Suzuki

Yutaka Suzuki

Junji Suzumiya

Sho Tabata

Takahiro Tabuchi

Hiroshi Tada

Hiroyuki Tagawa

Ayumu Taguchi

Keiko Taguchi

Ken Tajima

Tatsuro Tajiri

Masahiro Takada

Masatoshi Takagi

Satoshi Takagi

Akiko Takahashi

Kei Takahashi

Masanobu Takahashi

Masayuki Takahashi

Motoko Takahashi

Naoto Takahashi

Ryota Takahashi

Ryou‐u Takahashi

Satoru Takahashi

Tsuyoshi Takahashi

Yoshihisa Takahashi

Yoshinori Takahashi

Yuki Takahashi

Atsushi Takai

Tomoiku Takaku

Akiyoshi Takami

Hirokazu Takami

Kiyoko Takane

Toshimi Takano

Akinori Takaoka

Akifumi Takaori‐Kondo

Hiroki Takashima

Ryo Takata

Kenichi Takayama

Koichi Takayama

Tetsuji Takayama

Haruna Takeda

Masayuki Takeda

Tetsuo Takehara

Ichiro Takemasa

Katsuto Takenaka

Hideyuki Takeshima

Fumitaka Takeshita

Akinobu Taketomi

Hiroya Takeuchi

Shinji Takeuchi

Yasuto Takeuchi

Yuichi Takiguchi

Takahisa Takino

Jun Takizawa

Keiyo Takubo

Niidome Takuro

Satoshi Tamada

Yoshinori Tamada

Sho Tamai

Kentaro Tamaki

Mayumi Tamari

Hideto Tamura

Kenji Tamura

Nobuko Tamura

Yasuaki Tamura

Kazuaki Tanabe

Masahiko Tanabe

Atsushi Tanaka

Ichidai Tanaka

Kentaro Tanaka

Kozo Tanaka

Mariko Tanaka

Minoru Tanaka

Miwa Tanaka

Nobuyuki Tanaka

Shota Tanaka

Takuji Tanaka

Toshiyuki Tanaka

Yoshimasa Tanaka

Yoshio Tanaka

Yuting Tang

Shoichiro Tange

Akira Tangoku

Hiroaki Taniguchi

Hiroya Taniguchi

Naoyuki Taniguchi

Chizu Tanikawa

Keiji Tanimoto

Kenzui Taniue

Nobuhiro Tanuma

Yusuke Tarumoto

Etsu Tashiro

Satoshi Tashiro

Kensuke Tateishi

Ryosuke Tateishi

Hiroaki Tateno

Kenji Tatsuno

Isao Tawara

Hiroshi Tazawa

Jun Teishima

Naoki Terada

Hideki Terai

Shuji Terai

Takeshi Terashima

Yasuhito Terui

Masahiro Toda

Yosuke Togashi

Yuji Toiyama

Eriko Tokunaga

Fuminori Tokunaga

Shuta Tomida

Kei Tomihara

Akihiro Tomita

Hiroyuki Tomita

Naoto Tomita

Yoshihiko Tomita

Arata Tomiyama

Seiji Torii

Yukari Totsuka

Tatsuya Toyama

Takeshi Toyoda

Shinichi Toyooka

Yukari Tsubata

Shoma Tsubota

Katsuya Tsuchihara

Takahiro Tsuchikawa

Kiichiro Tsuchiya

Naoto Tsuchiya

Ryuto Tsuchiya

Hiroyuki Tsuda

Masumi Tsuda

Motoyuki Tsuda

Akihito Tsuji

Atsushi Tsuji

Tomohide Tsukahara

Hirotake Tsukamoto

Nobuo Tsukamoto

Tetsuya Tsukamoto

Kunihiro Tsukasaki

Tadasuke Tsukiyama

Ryouichi Tsunedomi

Masayuki Tsuneki

Takuya Tsunoda

Daisuke Tsuruta

Koji Tsuta

Sho Tsuyuki

Toyonori Tsuzuki

Jiancheng Tu

Ken Uchibori

Naoyuki Uchida

Shinya Uchino

Keiko Udaka

Masashi Ueda

Yoshihide Ueda

Satoshi Ueha

Arisa Ueki

Keisuke Ueki

Hiroji Uemura

Motohide Uemura

Sayaka Ueno

Takayuki Ueno

Hiroyuki Uetake

Hisashi Uhara

Shigeki Umemura

Hiroshi Uozaki

Fumihiko Urabe

Yuji Urabe

Takeshi Urano

Hiroshi Ureshino

Tetsuo Ushiku

Ryo Ushioda

Takeo Usui

Tatsuya Usui

Kensuke Usuki

Rogier Versteeg

Hisashi Wada

Keiko Wada

Satoshi Wada

Keiji Wakabayashi

Yuichi Wakabayashi

Kenji Wakai

Michihito Wakai

Bin Wang

Feng Wang

Hai‐Xia Wang

Yong Wang

Hideki Wanibuchi

Douglas G. Ward

Tadashi Watabe

Atsushi Watanabe

Keisuke Watanabe

Kentaro Watanabe

Kousuke Watanabe

Nobumoto Watanabe

Satoshi Watanabe

Takashi Watanabe

Tatsuro Watanabe

Toshiki Watanabe

Yukihide Watanabe

Richard Wong

Kongming Wu

Dongxi Xiang

Dawei Xu

Rong Xu

Tao Xu

Hiroshi Yagi

Tomonori Yaguchi

Hideaki Yahata

Hidetaka Yamada

Tadaaki Yamada

Tesshi Yamada

Hiroki Yamaguchi

Kiyoshi Yamaguchi

Motoko Yamaguchi

Yoshifumi Yamaguchi

Taiki Yamaji

Kazuhito Yamamoto

Kazuo Yamamoto

Keisuke Yamamoto

Masahiro Yamamoto

Mizuki Yamamoto

Seiji Yamamoto

Yuki Yamamoto

Yusuke Yamamoto

Yutaka Yamamoto

Kazuhiro Yamanoi

Kentaro Yamao

Shoji Yamaoka

Toshinari Yamasaki

Hiroharu Yamashita

Keishi Yamashita

Satoshi Yamashita

Taro Yamashita

Tatsuya Yamashita

Yo‐Ichi Yamashita

Takahiro Yamauchi

Hiroki Yamaue

Etsuko Yamazaki

Kentaro Yamazaki

Wei Yan

Teruki Yanagi

Hideyuki Yanai

Nozomu Yanaihara

Tomonori Yano

Masakazu Yashiro

Hiroyuki Yasuda

Jun Yasuda

Takahiko Yasuda

Ryuji Yasumatsu

Yota Yasumizu

Jun‐Ichirou Yasunaga

Masahiro Yasunaga

Takuya Yazawa

Chi‐Tai Yeh

Gang Yin

Takehiko Yokobori

Masanao Yokohira

Akihiko Yokoyama

Satoru Yokoyama

Hiroshi Yokozaki

Sho Yoneda

Toshiyuki Yoneda

Kan Yonemori

Kimio Yonesaka

Kenichi Yoshida

Kiyotsugu Yoshida

Noriaki Yoshida

Tatsuya Yoshida

Kosuke Yoshihara

Takako Yoshii

Tetsuro Yoshimaru

Yasuhiro Yoshimatsu

Akihide Yoshimi

Koji Yoshimoto

Yuki Yoshino

Yusuke Yoshioka

Hideyuki Yoshitomi

Hironori Yoshiyama

Akihiko Yoshizawa

Tomoharu Yoshizumi

Danny R. Youlden

Qiang Yu

Zuoren Yu

Takeshi Yuasa

Masayoshi Zaitsu

Yoshitaka Zenke

Biyuan Zhang

Caiguo Zhang

Chaoqi Zhang

Jing Zhang

Yi Zhang

Shuliang Zhao

Xiaogang Zhao

Ming Zhou

Han Zou

Youli Zu

